# Roles of Lipids in the Permeability Barriers of Skin and Oral Mucosa

**DOI:** 10.3390/ijms22105229

**Published:** 2021-05-15

**Authors:** Philip W. Wertz

**Affiliations:** University of Iowa, Iowa City, IA 52242, USA; philip-wertz@uiowa.edu; Tel.: +1-319-337-4364

**Keywords:** barrier function, ceramides, cholesterol, fatty acids, intercellular lamellae, keratinocytes, oral mucosa, skin

## Abstract

PubMed searches reveal much literature regarding lipids in barrier function of skin and less literature on lipids in barrier function of the oral mucosa. In terrestrial mammals, birds, and reptiles, the skin’s permeability barrier is provided by ceramides, fatty acids, and cholesterol in the outermost layers of the epidermis, the stratum corneum. This layer consists of about 10–20 layers of cornified cells embedded in a lipid matrix. It effectively prevents loss of water and electrolytes from the underlying tissue, and it limits the penetration of potentially harmful substances from the environment. In the oral cavity, the regions of the gingiva and hard palate are covered by keratinized epithelia that much resemble the epidermis. The oral stratum corneum contains a lipid mixture similar to that in the epidermal stratum corneum but in lower amounts and is accordingly more permeable. The superficial regions of the nonkeratinized oral epithelia also provide a permeability barrier. These epithelial regions do contain ceramides, cholesterol, and free fatty acids, which may underlie barrier function. The oral epithelial permeability barriers primarily protect the underlying tissue by preventing the penetration of potentially toxic substances, including microbial products. Transdermal drug delivery, buccal absorption, and lipid-related disease are discussed.

## 1. Introduction

Several reviews of various aspects of the roles of lipids in the barrier function of the skin have been published recently [1,2,3,4]. Major points will be summarized here, but the readers are referred to these previous reviews for more detail.

The surface of an average human skin is approximately two square meters in area and serves as our interface with the environment. It serves to prevent loss of water and electrolytes. It also effectively prevents the penetration of a range of potentially harmful chemicals from the environment, including microbial products. The evolution of life on dry land required the development of watertight skin [5]. The permeability barrier of the skin is provided by the outermost layer, the stratum corneum [6]. Specifically, an approximately equimolar mixture of ceramides, fatty acids, and cholesterol between the flattened, cornified cells determine the permeability of the skin [7].

The situation in the oral cavity has some similarities to the skin, but there are also some distinct differences. The oral mucosa is coated with saliva, so there is no water gradient. Therefore, there is no driving force for water loss through the mucosa. Nevertheless, there is a need for a barrier sufficient to prevent the penetration of potentially harmful substances from food, beverages, and microorganisms. Lipids appear to play some role in the oral mucosal barriers; however, this has been much less studied than the barrier lipids of the skin. Removal of lipids from buccal mucosa resulted in a marked increase in permeability, suggesting a role for lipids in barrier function of nonkeratinized oral epithelia [8]. Much of the works on the oral mucosal barriers have been directed at the delivery of drugs by absorption through the mucosa of the mouth [9].

In the barriers of both skin and oral mucosa, substances that penetrate the barrier do so by passive diffusion [10,11]. The primary pathway through which compounds diffuse through these permeability barriers is paracellular [12,13]. Sebaceous follicles of the skin have been shown to serve as a shunt pathway [14]. Although there are sebaceous glands, sometimes called Fordyce granules, throughout the oral mucosa their significance in drug uptake is uncertain [15,16]. Likewise, sweat ducts and salivary ducts are potentially minor, and relatively unexplored, potential routes by which substances could cross the skin or mucosa, respectively.

## 2. Skin

Nicolaides identified ceramide as a skin lipid in 1965 based on an infrared spectrum of polar material recovered from the origin of a thin-layer chromatographic plate [17].

The work of G.M. Gray and associates in the mid to late 1960s established ceramides as major lipids of the permeability barrier [18]. This group established that the lipid composition of epidermal keratinocytes alters dramatically as a function of differentiation [19]. The basal and spinous cells contained mostly phosphoglycerides and sphingomyelin with a small amount of cholesterol. As differentiation proceeds, the amount of lipid increases with the amounts per cell of both phospholipids and cholesterol increasing, but glucosylceramides also accumulate. Near the boundary of the granular layer and the stratum corneum phospholipids are broken down. Fatty acids are released from the phosphoglycerides, and ceramides are released from sphingomyelin. Glucosylceramides are deglycosylated to produce ceramides. Ceramides, fatty acids, and cholesterol are the main lipids in the stratum corneum. The fatty acids in the stratum corneum are mainly saturated, range from 14 through 28 carbons in length, and the most abundant are C22:0 and C24:0 [20].

It was established that the glucosylceramides and ceramides are structurally heterogeneous [18]. Although individual ceramide structures were not elucidated, most of the building block fatty acids and long-chain bases were identified. The fatty acids included normal and α-hydroxyacids both ranging between 14- and 30-carbons in length. The 24- and 26-carbon saturated normal and α-hydroxyacids were prominent. The long-chain bases were from 16- through 22-carbons in length, and included sphingosines, dihydrosphingosines, and phytosphingosines. The major species identified were 18-carbon entities, and some unidentified entities were noted.

The most abundant and least polar of the glucosylceramides was isolated, and the structure was partially identified [21]. The β-glycosidically attached glucose was identified by proton magnetic resonance of the intact molecule and by gas-liquid chromatography of the trimethylsilalated isolated sugar. The major ester-linked fatty acid was identified as an 18-carbon diene, probably linoleic acid by gas-liquid chromatography of the methyl esters. It constituted 77.4 % (pig) to 56.2% (human) of the total fatty acids. The long-chain bases were shown to consist of a mixture of sphingosines and dihydrosphingosines by gas-liquid chromatography of the trimethylsilated derivatives. However, the ester-linked fatty acids were said to be mainly attached to the 3-hydroxyl group of the glucose, and based on electron impact mass spectral data, the amide-linked fatty acid was said to be 35-carbons long with two double bonds and two hydroxyl groups between carbon-16 and carbon-20. These latter two features subsequently proved to be incorrect. The amide-linked fatty acid was subsequently identified as a series of mostly 30- through 34-carbon ω-hydroxyacids, and the identity of the 18-carbon diene as linoleic acid was confirmed [22]. The ester-linked fatty acid was found to be attached to the ω-hydroxyl group of the amide-linked hydroxyacid [23,24].

**Figure 1:**
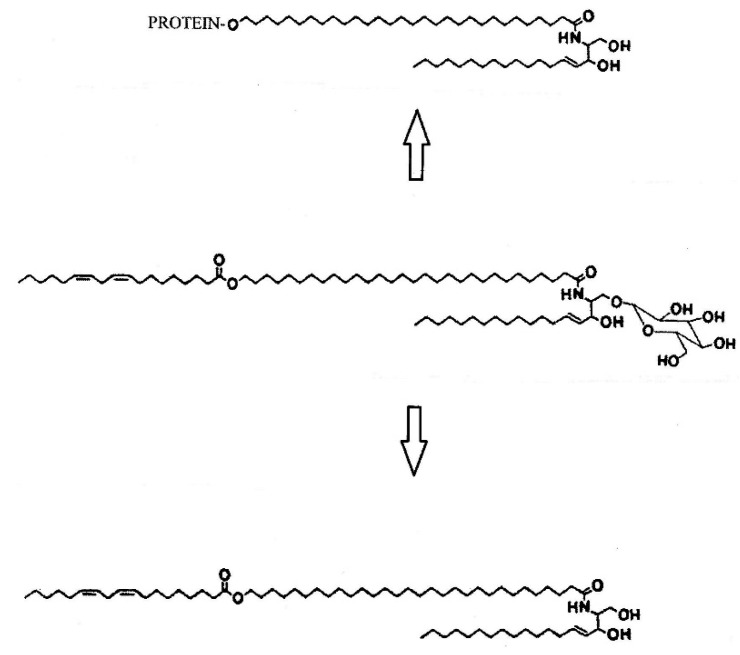
The linoleate-rich acylglucosylceramide (**center**) is the precursor of the linoleate-rich acylceramide (**bottom**) found in the intercellular spaces of the stratum corneum and the covalently bound ω-hydroxyceramide (**top**), or CLE.

During the 1980s the structures of the glucosylceramides and ceramides from pig epidermis were published [25,26]. The ceramides included an ω-O-acylceramide analogous to the acylglucosylceramide [24,26]. Isolated epidermal lamellar granules were shown to be enriched in glucosylceramides, including the linoleate-containing acylglucosylceramide [27,28]. Acylglucosylceramide in the bounding membrane of the lamellar granules is the precursor of the ω-hydroxyceramides that become attached to the outer surface of the cornified envelope, thereby forming the corneocyte lipid envelope (CLE) [4,29,30]. Acylglucosylceramide within the internal lamellae of the lamellar granule becomes deglycosylated to form the acylceramide that passes into the intercellular spaces of the stratum corneum [24,26]. This is illustrated in Figure 1.

Transmission electron microscopy with ruthenium tetroxide post-fixation revealed multilamellar structures within the intercellular spaces of the stratum corneum [31]. Multiple trilamellar repeat units, each of which, had a 13 nm overall dimension were evident. This same 13 nm repeat unit was found by X-ray diffraction, and it was shown that the linoleate-containing acylceramide was required for the formation of this 13 nm trilamellar structure [32,33,34].

Both X-ray diffraction and electron diffraction reveal that the lateral packing of lipids in healthy stratum corneum is predominantly orthorhombic [35,36,37,38]. Upon heating, there is a shift from orthorhombic lateral packing to hexagonal packing. Moreover, in atopic dermatitis and lamellar ichthyosis, where barrier function is impaired, hexagonal packing is increased relative to orthorhombic packing [39]. In lamellar ichthyosis, hexagonal packing predominates.

**Figure 2:**
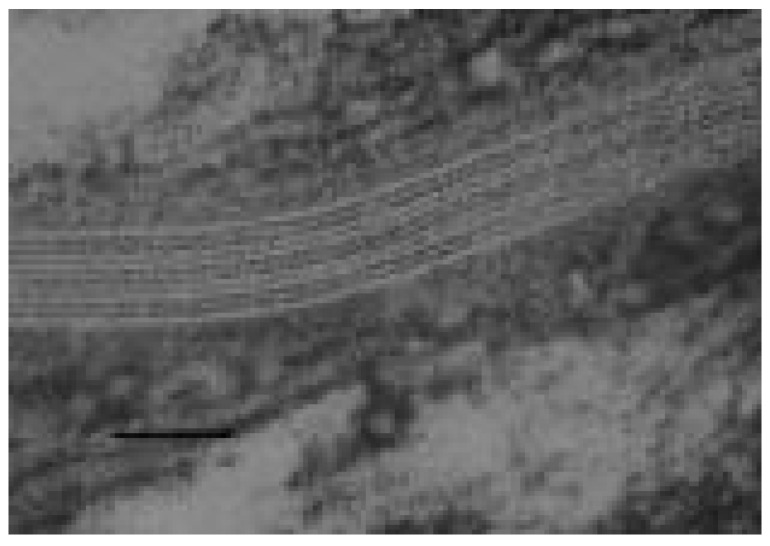
Transmission electron micrograph of RuO_4_-fixed human stratum corneum showing intercellular lamellae between two corneocytes (SC). The first lucent band on either side of the intercellular space is the CLE. Bar = 50 nm.

**Figure 3:**
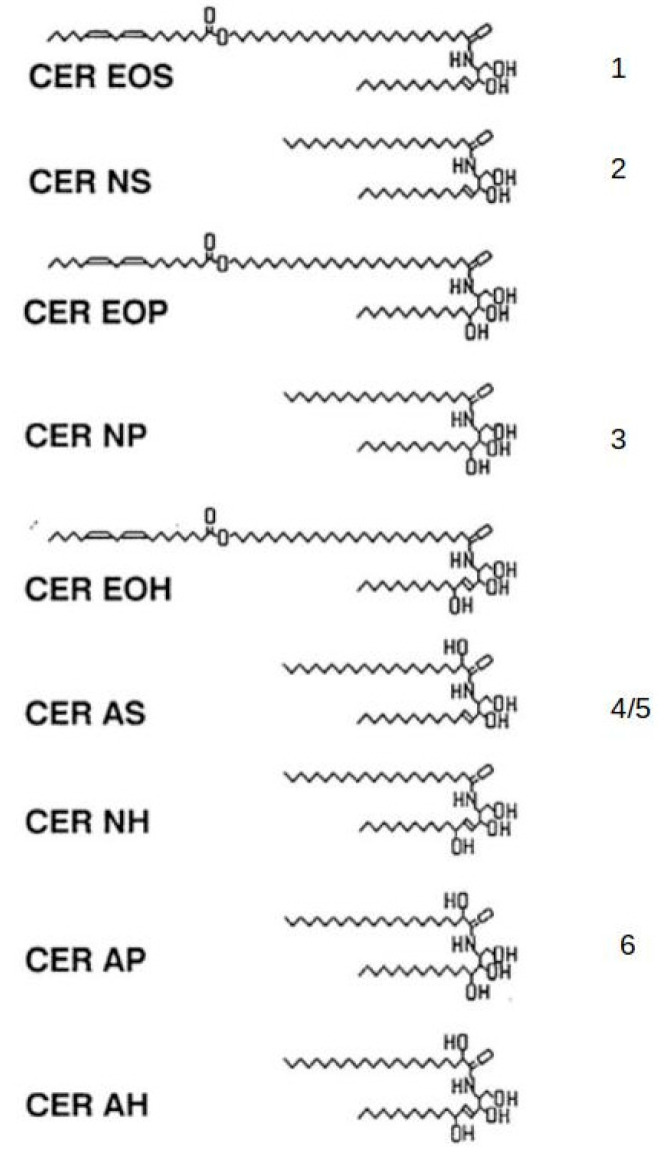
Structures of ceramides from human stratum corneum [47]. In the nomenclature on the left S, P, and H indicate sphingosine, phytosphingosine and 6-hydroxysphingosine, and N, A, and O indicate normal fatty acid, α-hydroxyacid and ω-hydroxyacid. E indicates the presence of an ester-linked fatty acid [48]. In these structures, sphingosine is always accompanied by dihydrosphingosine. The numbers on the right indicate the ceramides found in the porcine stratum corneum [26]. In this case, CER AS (4/5) separates into two fractions on thin-layer chromatography. Fraction 4 contained mainly C24–C28 α-hydroxyacids, while fraction 5 contained α-hydroxypalmitic acid as the sole amide-linked fatty acid.

Lamellar granules bud off from a tubuloreticular membrane system in the outermost granular cells [40]. At the boundary between the granular layer and the stratum corneum, the bounding membrane of the lamellar granule fuses into the cell plasma membrane, and the contents of the granule are extruded into the intercellular space [41]. At this time, glucocerebrosidase converts glucosylceramide to ceramides, and acid sphingomyelinase converts sphingomyelin to ceramide [42,43]. Fatty acids are released from phosphoglycerides [44,45]. This lipid remodeling is accompanied by fusion of the short lamellae to form broad multilamellar sheets [46], shown in Figure 2. Acylglucosylceramide from the lamellar granule gives rise to the CLE and the acylceramide among the free lipids in the stratum corneum. Together, the CLE and intercellular lipid lamellae determine the permeability of the skin. The structures of human stratum corneum-free ceramides are shown in Figure 3.

## 3. Keratinized Oral Mucosa

All regions of the oral mucosa are covered by a stratified squamous epithelium; however, the pattern of differentiation varies regionally [49]. The gingiva and hard palate are covered by a keratinizing epithelium similar to the epidermis of the skin. The floor of the mouth, buccal regions, and the underside of the tongue are covered by a nonkeratinized epithelium. The specialized epithelium on the dorsum of the tongue is approximated as a mosaic of keratinized and nonkeratinized epithelia.

In the keratinized oral epithelia, lipids are packaged in lamellar granules just as in the epidermis [50,51]. However, the volume density of lamellar granules in the keratinizing oral epithelia is less than in the epidermis [52]. Accordingly, the lipid content of the oral stratum corneum is less than that in the epidermal stratum corneum [53]. The stratum corneum from the gingiva and hard palate contain ceramides, cholesterol, and fatty acids, but they also contain small amounts of phospholipids that are not found in the epidermal stratum corneum The ceramide profile of the oral stratum corneum was similar to that of the epidermal stratum corneum. A more detailed analysis of the ceramides from the porcine palatal stratum corneum revealed that the structures were almost identical to the structures determined for porcine epidermal ceramides shown in Figure 2 [54]. The one exception was that while linoleate is the major (71.7%) ester-linked fatty acid in epidermal ceramide EOS, in the palatal ceramide EOS linoleate is only 8.8% of the ester-linked fatty acids. The other ester-linked fatty acids were C16:0, C18:0, C20:0, and C18:1. Linoleate-rich ceramide EOS is thought to be essential for the formation of the 13-nm trilamellar repeat units in the epidermal stratum corneum intercellular lipids [33,55,56]. In Figure 1, there are nine lucent bands in the intercellular space, and these bands appear to alternate broad-narrow-broad in width. Each of these trilayers represents one 13 nm repeat unit. The modified ceramide EOS found in the keratinized oral epithelium does not support the formation of these trilamellar structures [57]. Paired bilayers and sometimes swirls of lamellae are seen in electron micrographs.

The fatty acid composition of the palatal ceramide EOS has implications for the CLE. In the epidermis, the precursor to ceramide EOS is a glucosylceramide, and both have identical fatty acid compositions dominated by linoleic acid. The epidermal acylglucosylceramide is the precursor for both the acylceramide and the CLE. The palatal linoleate-poor ceramide EOS does not support the formation of the 13 nm trilamellar units, and its precursor does not support the formation of the corneocyte lipid envelope [58].

Because the oral stratum corneum contains less lipid than the epidermal stratum corneum, and because the lipid in the oral tissue is less orderly organized, it would be expected that the permeability of the keratinized oral regions would be greater than that of skin. This is the case [59]. The permeability coefficient for tritiated water, Kp, was approximately ten times higher for palatal tissue compared to skin.

## 4. Nonkeratinized Oral Mucosa

The nonkeratinizing oral mucosal regions do not have an anatomically distinct barrier. In the keratinizing epithelia, the internal organelles are degraded during differentiation, and the plasma membrane becomes replaced by a thick band of olymerized protein. In the nonkeratinizing regions, the internal organelles and plasma membrane are retained. This makes it difficult to study roles for lipids in the barrier function of these regions. The superficial layers of these nonkeratinized epithelia can be isolated by tryptic digestion, and the lipids can be extracted and analyzed [53]. However, most of the lipid so obtained comes from the plasma membrane and residual internal organelles. It is not possible to determine which lipids are involved in barrier function from the analysis of such extracts. However, there is some ceramide (CER NP), and abundant cholesterol and free fatty acids are present. This suggests the possibility that there may be a barrier somewhat resembling that found in the keratinizing epithelia.

**Figure 4:**
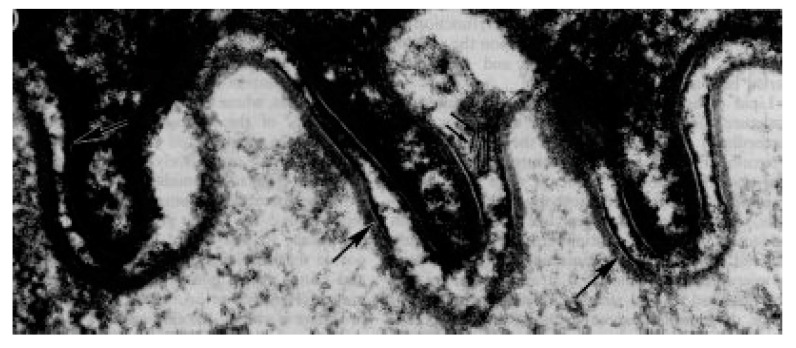
Transmission electron micrograph of RuO4-fixed superficial porcine epithelium from the floor of the mouth. The arrow indicates the plasma membrane. The double arrows indicate a stack of lamellae running transversely across the intercellular space. The magnification was ×100,000. Reprinted with permission from ref. [53]. Copyright 1995 Elsevier.

The epithelium from the floor of the mouth is, on average, 192 µm thick, while the buccal epithelium is 772 µm thick [60]. When horseradish peroxidase or lanthanum were used to follow the movement of water through these tissues it was observed that neither agent could penetrate from the surface of the tissue; however, both could freely penetrate between the cells when introduced from the underside of the tissue. Progress of these penetrants halted at approximately two-thirds of the way from the bottom to the surface [60,61,62]. Thus, it appears that this superficial outer third of the epithelium represents the permeability barrier. This localization corresponds to the location at which “membrane-coating granules” discharge their contents [50,63]. These membrane-coating granules are of about the same size as the lamellar granules seen in the epidermis and keratinized oral epithelia; however, instead of lamellar material, the granules contain an electron-dense core with electron-dense filaments extending toward the bounding membrane. The chemical nature of this material is unknown. In 1995, a second secretory vesicle of similar size to the cored granules was reported [53]. This secretory granule was not apparent in transmission micrographs of samples prepared with conventional osmium tetroxide fixation. It was only seen in sections of specimens fixed with ruthenium tetroxide. These granules contained lamellar material and have been called lamellate granules. Their content is discharged near the bottom of the superficial layer of the epithelium and can be seen as short stacks of lamellae running diagonally across the intercellular space, as illustrated in Figure 4.

## 5. Transdermal Drug Delivery

Most drugs are administered orally as pills or liquids or by injection. The transdermal route of drug delivery has several advantages over the oral and parenteral routes of drug delivery. The skin is readily accessible and avoids the acid environment of the stomach, the digestive enzymes of the gut, and first-pass metabolism in the liver. Unlike swallowing a pill or receiving an injection, transdermal drug delivery patches can deliver the drug over an extended period. This can maintain a relatively constant systemic concentration of the drug. Should one develop an adverse drug reaction, the patch can be removed. One disadvantage of the transdermal patch is that some individuals develop irritation at the site of patch placement. There are also limitations to the kinds of molecules that can be delivered through the skin [64]. Molecules with molecular weights (MW) greater than about 350 daltons do not penetrate well. Additionally, the polarity of the molecule must fall within a limited range. Polarity is quantitatively assessed as an oil-water or octanol-water partition coefficient. Generally, the LOG (K_octanol-water_) must fall within the range of about 1 to 4 [64]. Table 1 lists several drugs that are delivered via transdermal patches. Compounds with lower octanol-water partition coefficients cannot partition into the intercellular lipids of the stratum corneum. Compounds with higher octanol-water partition coefficients can enter the stratum corneum but are too hydrophobic to enter the more aqueous environment of the viable epidermis.

Many efforts have been made to enhance the flux of drugs through the stratum corneum [65]. A wide range of chemicals has been tested in this regard. Among the most widely used chemical permeability enhancers are ethanol, unsaturated fatty acids, surfactants, and terpenoids. Flexible liposomes prepared with phospholipids and surfactant were once thought to transport encapsulated drugs across the stratum corneum by squeezing through the intercellular spaces; however, it is now thought that the surfactant permeabilizes the intercellular lamellae to enhance drug flux [66]. There may also be increased hydration of the stratum corneum, which would increase permeability.

A variety of physical methods have also been used to increase drug flux across the stratum corneum. One of these methods is sonophoresis [67,68,69]. In this technique, low-frequency ultrasound increases the vibrational energy of the lipid molecules in the stratum corneum, thereby causing fluidization and increased permeability. This technique is capable of delivering macromolecules [70]. A disadvantage of this technique is that because of the required equipment its use is largely confined to hospitals.

Another technique that is used to increase skin permeability is iontophoresis [71]. In iontophoresis, a mild current is generated between a cathode and an electrode on the skin surface. Positively charged drugs, such as lidocaine hydrochloride, can be driven into the stratum corneum from a solution at the anode site. This electromigration is caused by electrostatic repulsion. Neutral molecules will flow with the current from anode to cathode by electro-osmosis. The apparatus for iontophoresis has been miniaturized to the size of a wristwatch [72]. The GlucoWatch Biographer (Cygnus Inc., Redwood City, CA, USA), brought to market in 2002, used electro-osmosis to sample glucose from interstitial fluid for diabetic monitoring [73]. Unfortunately, this device did not meet expectations and was taken off the market in 2007 [74]. The most common use of iontophoresis is the treatment of palmar or plantar hyperhidrosis [75]. This is frequently done with tap water so that hydrogen ions are delivered into the eccrine sweat glands, but sometimes anticholinergic agents are added.

Electroporation uses high voltage pulses to perturb the organization of the intercellular lipid lamellae of the stratum corneum [76]. Electroporation is a safe and effective method for cutaneous and subcutaneous delivery of bleomycin or cisplatin for the treatment of malignant melanoma or other malignancies [77].

Perhaps the most promising technology for bypassing the barrier of the stratum corneum is microneedles [78]. Microneedle array patches have been under development for several decades. Early difficulties with manufacturing methodology and properties of the product have finally been overcome, and at least eleven products are now on the market [79]. The microneedles are long enough to traverse the stratum corneum, which causes no pain. They can be solid, hollow, or dissolvable. Solid microneedles are coated with the active agent. while the active agent is pumped through the hollow microneedles. Dissolvable microneedles have the active agent incorporated into the needle which is usually a sugar or dissolvable protein or polymer [80]. The advent of 3D printing now facilitates the manufacturing process [81]. By physically and painlessly breaching the barrier, there are no restrictions on what can be delivered across the barrier. Nucleic acids, proteins such as insulin, and ionic materials can now cross the skin barrier. The skin is an attractive site for vaccination. Because of the Langerhans cells in the epidermis and the dendritic cells in the dermis, the skin would appear to be a better site for vaccination than muscle which lacks antigen-presenting cells [82,83]. This is supported by an animal experiment in which the results of microneedle array delivery of influenza vaccine to the skin were compared to intramuscular vaccination [84]. When compared to the intramuscular group, the microneedle group had more influenza-specific antibody-producing cells. In addition, there was increased activation of helper T cells and formation of germinal centers in regional lymph nodes.

## 6. Skin Disease and the Barrier

Poison ivy rash is classic allergic contact dermatitis and an occupational hazard for outdoor workers [85]. It is caused by a mixture of phenolic compounds known collectively as urushiol [86]. Urushiols consist of catechol (1,2-dihydroxybenzene) with a linear carbon chain attached to carbon 3. The side chains all contain 15 carbon atoms, but they differ in the number (0–3) and location of double bonds. All have molecular weights in the range of 314–320 daltons. Although no published octanol-water partition coefficient could be found, these molecules readily cross the stratum corneum barrier.

Atopic dermatitis is a skin disease that, although it can occur at any age, usually presents in early childhood. It is characterized by dry, itchy, and easily irritated skin with fluctuating disease activity. Many atopic subjects also suffer from asthma. Transepidermal water loss is elevated indicating impaired barrier function [87]. It has long been known that stratum corneum lipids are altered in this condition [88,89,90,91]. In general, when comparing lipids from the atopic stratum corneum to the lipids from the normal control stratum corneum, the total amount of ceramide was reduced. The ceramide to cholesterol ratio was reduced, and some ceramide fractions were altered more than others. The linoleate containing ceramide EOS was significantly lower in the atopic stratum corneum, and oleate replaced some of the linoleate [89,91]. The reduced level of ceramides in atopic dermatitis is at least partly the result of an elevated enzyme activity that can hydrolyze the amide linkages in both sphingomyelin and glucosylceramide [92]. The products of this reaction can no longer be converted into ceramides. More recently, the free fatty acids and ceramides from the atopic stratum corneum have been compared in detail to those from the normal control stratum corneum [93]. The free fatty acids from the atopic subjects were shifted toward shorter chain lengths and contained more monounsaturated species. Likewise, the ceramides contained, on average, shorter fatty acids. When compared by ATR-FTIR, the atopic stratum corneum showed less lateral order in lipid packing compared to the control stratum corneum. Less orderly chain packing would be expected to lead to increased transepidermal water loss, as is seen in this condition. Similar observations have been made on the free fatty acids and ceramides in the stratum corneum of Netherton syndrome [94]. The skin of subjects with atopic dermatitis tends to become colonized with *Staphylococcus aureus* [95]. This represents a risk for infection and may reflect the fact that both free sphingosine and sapienic acid are below normal levels in atopic skin [96,97]. Both of these lipids are potent antimicrobials against *S. aureus* [98].

Psoriasis is a relatively common inflammatory skin disease that affects over 60 million people worldwide [99]. Psoriasis has several clinical presentations; however, the most common type is plaque psoriasis or psoriasis vulgaris. Psoriasis can occur at any age in either gender. Plaque psoriasis is characterized by pink plaques with silvery scales on white skin or grey plaques on black skin. The plaques are hyperproliferative and have elevated transepidermal water loss [100]. The proportions of the ceramides from the psoriatic scale are altered compared to normal with a reduction of ceramide EOS being most notable [48,101,102,103]. In one study, the levels of serinepalmitoyl transferase (SPT), the rate-limiting enzyme in ceramide synthesis, and ceramidase in psoriatic scale and nonlesional skin were compared [104]. The level of SPT was lower in the psoriatic scale compared to the uninvolved stratum corneum, but there was no difference in the ceramidase levels. Since ceramides are signaling molecules that can slow proliferation, it was suggested that the reduced ceramide level may be responsible for the hyperproliferation. The levels of free sphingosine and dihydrosphingosine were found to be higher on the psoriatic scale compared to stratum corneum from uninvolved skin. This may reflect excess long-chain base synthesis rather than ceramidase action. Since the long-chain bases are potent antimicrobials this finding may help to explain the fact that psoriatics do not suffer many skin infections compared to atopics [98,105].

Mutations in several genes that catalyze steps in the biosynthesis of the corneocyte lipid envelope cause various autosomal recessive congenital ichthyoses, or ARCI [3,4]. These include *ELOV1*, *ELOV4*, *CER3*, *PNPLA1*, *ABHD5*, *FATP4*, *12R-LOX*, *ALOXE3*, *CYP4F22*, *SDR9C7*, and *TGM1*. *ELOV1* and *ELOV4* are fatty acyl-CoA elongase genes. Mutations in these elongase genes not only result in ARCI but also result in neurologic malfunction. *CER3* codes for a ceramide synthase. *FATP4* codes for an enzyme that converts long-chain ω-hydroxyacids to the corresponding CoA thioester. *PNPLA1* codes for an enzyme that transfers the linoleate from a triglyceride or phosphoglyceride to the ω-hydroxyl group of the ω-hydroxyacid, and the product from *ABHD5* is a cofactor of the *PNPLA1* product. Mutations in *ABHD5* cause ARCI and Chanarin-Dorfman syndrome, a lipid storage disease [106]. The ω-hydroxylation is mediated by the *CYP4F22* gene product [107]. 12RLOX and eLOX3, the enzyme coded for by *ALOXE3*, modify the linoleate before the attachment of lipid to the cornified envelope [108]. *TGM1* codes for transglutaminase 1, which is essential in the formation of the cornified envelope and may attach the ω-hydroxyceramide to the outer surface of the cornified envelope to form the corneocyte lipid envelope [109]. Mutations in this gene result in lamellar ichthyosis [110]. SDR9C7 provides an alternative to transglutaminase-mediated attachment of lipid to the cornified envelope to form the CLE [111]. The importance of these genes in the biosynthesis of the CLE has been confirmed by a series of experiments using knockout mice [3].

Mutations of *ABCA12* result in a severe form of ARCI, Harlequin ichthyosis [112]. Newborns with Harlequin ichthyosis are covered with a thick membrane with large diamond-shaped plates. In this condition, approximately 50% mortality is seen in the perinatal period. After shedding this initial collodion membrane, the affected subjects have congenital ichthyosiform erythroderma (CIE), a red scaly skin. The gene product of *ABCA12* is an ATP binding cassette transporter necessary for transferring lipid into the lamellar granules that then deliver this lipid to the bottom of the stratum corneum. In Harlequin ichthyosis, the “empty” lamellar granules are seen by transmission electron microscopy, and a corneocyte lipid envelope is present [3]. However, the intercellular spaces of the stratum corneum contain only sparse lamellar material. CIE subjects have much elevated transepidermal water loss, and dehydration can be a problem.

Recessive X-linked ichthyosis (RXLI) is characterized by dry scaly skin with elevated transepidermal water loss [113]. Cholesterol sulfate was identified as a minor component of epidermal lipids in 1975 [19]. This type of ichthyosis was first recognized as being sex-linked by Wells and Kerr [114]. It was subsequently shown to be due to a sterol sulfatase deficiency [115]. In normal epidermis, cholesterol sulfate represents about 5–10% of the polar lipid or about 2–3% of the total lipid; however, it is present in only trace amounts in exfoliated material [116,117]. In the stratum corneum from RXLI, cholesterol sulfate accumulates to about six times the normal concentration [118]. Cholesterol sulfate inhibits serine proteases that normally would degrade corneodesmosomes leading to desquamation [119]. This inhibition of desquamation accounts for the scaliness in RXLI. It has been suggested that excess cholesterol sulfate in the stratum corneum could induce a lamellar to a nonlamellar lipid phase transition, which could account for the defective barrier [120].

## 7. Buccal Absorption

The nonkeratinized oral regions of the oral mucosa are more permeable than the keratinized regions making the floor of the mouth and underside of the tongue and the buccal regions more attractive for drug delivery. In fact, for more than a century nitroglycerin has been delivered systemically to alleviate angina pain by placement under the tongue [121]. The floor of the mouth is the most permeable region of the oral mucosa, and accordingly is the most frequent site of oral squamous cell carcinoma because this is where the carcinogens can best penetrate. However, the buccal regions provide greater surface area and, therefore, have received more attention regarding drug delivery. These attempts usually involve a drug incorporated into an ointment, gel, or mucoadhesive patch. Colgate-Palmolive markets Orabase with benzocaine for pain relief. The most common oral film materials for transmucosal drug delivery include the polysaccharide polymer pullulan, maltodextrin, alginate, pectin, chitosan, and other natural polymers [122].

Many of the chemical permeation enhancers identified for skin have proven to also enhance buccal permeability [123,124]. These include surfactants, bile salts, fatty acids, ethanol, terpenes, and azone. Interestingly, one of the commonly used film materials, chitosan, is a permeability enhancer [124]. Chitosan is partially deacetylated chitin from shellfish. Chitin is a linear polymer of N-acetylglucosamine in β-(1→4) linkage. At physiological pH, chitosan is positively charged. In one study, chitosan was compared with negatively charged sulfobutyl ether-β-cyclodextrin and neutral hydroxypropyl-β-cyclodextrin as permeability enhancers for transbuccal delivery of ropinirole hydrochloride [125]. All three polymers enhanced permeability to the test drug with chitosan being the most effective hydroxypropyl-β-cyclodextrin being least effective. An FTIR study of the treated tissue indicated alteration of lipid organization with possible solubilization of the intercellular lipid. The symmetric and asymmetric methylene stretching absorbance were broadened and diminished in the area by the neutral and negatively charged polymers, but these signals completely disappeared in the tissue treated with chitosan.

The buccal drug delivery route permits the delivery of much larger molecules than skin. For transdermal delivery, the molecular weight cutoff is about 350 daltons. In a study of diffusion of fluorescein-conjugated dextrans through the porcine buccal mucosa, the molecular weight cutoff was somewhere between 10,000 and 20,000 daltons [126]. This raises the possibility of delivering peptides and nucleic acid via this route. More than thirty studies have been directed at various aspects of delivering insulin systemically by buccal absorption. A variety of methodology has been used, including various films, penetration enhancers, and incorporation of insulin into nanoparticles. One of the more unusual approaches used a conjugate of a cell-penetrating peptide (LMWP) with pegylated insulin (INS-PEG-LMWP) [127]. The uptake and transportation of INS-PEG-LMWP across buccal mucosa were much enhanced compared to insulin in solution. In a rabbit model, the insulin derivative had good bioavailability and effectively reduced circulating glucose levels. An earlier study incorporated insulin and a cell-penetrating peptide into nanoparticles which enhanced insulin delivery across a mucous secreting cell culture [128]. A mucoadhesive patch containing these nanoparticles was shown to increase serum insulin concentration and induce a hypoglycemic response in diabetic rats. Another novel approach to enhance buccal absorption of insulin involved the use of an ionic liquid as a permeability enhancer [129]. Insulin was dissolved in the ionic liquid consisting of choline and geranic acid. This eutectic liquid was sandwiched between two layers of a mixed poly-(vinyl alcohol)/chitosan film. This increased the transport of insulin across porcine buccal mucosa sevenfold. When such patches were placed in the rat buccal pouch, blood glucose levels were reduced in a dose-dependent manner with up to a 50% drop recorded. Ionic liquids are unusually good solvents. In this case, the mechanism of action may be the extraction of lipids from the outer epithelium into a pool of ionic lipid at the mucosal surface [129].

In one innovative study, a combination of oxaliplatin-loaded chitosan nanoparticles combined with iontophoresis was tested as a possible means of treatment of tumors within the oral mucosa [130]. The mucoadhesive chitosan nanoparticles delivered an initial burst of drug followed by a longer-term steady release of the drug. The steady-state rate of drug penetration into porcine buccal mucosa was three times greater than that from oxaliplatin solution. This was further enhanced two-fold by iontophoresis.

Like skin, the buccal mucosa is an attractive site for immunization. One study tested the effectiveness of a microneedle array for immunization via murine buccal mucosa [131]. Solid microneedles in an array were coated with ovalbumin, and the microneedle array was applied to murine buccal mucosa twice two weeks apart. A high serum IgG titer was subsequently observed, thus validating this approach [132].

## Figures and Tables

**Table 1:** Molecular weights (MW) and LOG (K_octanol-water_) of several drugs that are delivered via transdermal patches. The data for this table were obtained from the National Library of Medicine through Pubchem (https://pubchem.ncbi.nlm.nih.gov, (accessed on 19 March 2021)).

Drug	MW	Log (K)
nicotine	162	1.2
nitroglycerin	227	1.6
lidocaine	234	2.3
estradiol	272	4.0
testosterone	288	3.3
scopolamine	303	1.0
fentanyl	336	4.1

## Data Availability

Not applicable.
